# Using Quantized Breakdown Voltage Signals to Determine the Maximum Electric Fields in a Quantum Hall Effect Sample

**DOI:** 10.6028/jres.100.019

**Published:** 1995

**Authors:** M. E. Cage, C. F. Lavine

**Affiliations:** National Institute of Standards and Technology, Gaithersburg, MD 20899-0001

**Keywords:** breakdown, electric fields, quantized dissipation, quantized voltage states, quantum Hall effect, quasi-elastic inter-Landau level scattering, two-dimensional electron gas

## Abstract

We estimate the maximum values of the electric field across the width of a GaAs/AlGaAs heterostructure quantum Hall effect sample at several currents when the sample is in the breakdown regime. This estimate is accomplished by measuring the quantized longitudinal voltage drops along a length of the sample and then employing a quasielastic inter-Landau level scattering (QUILLS) model to calculate the electric field. We also present a pictorial description of how QUILLS transitions occurring between states distributed across the sample width can be detected as voltage signals along the sample length.

## 1. Introduction

In the integer quantum Hall effect [1–3] the Hall resistance *R*_H_ of the *i* th plateau of a fully quantized two-dimensional electron gas (2DEG) has the value *R*_H_(*i*) = *h*/(*e*^2^*i*), where *h* is the Planck constant, *e* is the elementary charge, and *i* is an integer. The current flow within the 2DEG is nearly dissipationless in the Hall plateau regions of high-quality devices, and the longitudinal voltage drops, *V_x_*, along the sides of the sample are very small. At high currents, however, energy dissipation can suddenly appear in these devices [4,5] and *V_x_* can become quite large. This is the breakdown regime of the quantum Hall effect.

The dissipative breakdown voltage *V_x_* can be detected by measuring voltage differences between potential probes placed on either side of the device in the direction of current flow. Cage et al. [6–9] have found that these breakdown voltages can be quantized. We will use this quantization phenomenon, and a black-box model that is based on the conservation of energy, to determine the fraction of electrons making transitions between Landau levels and their transition rates [7–9]. We will also use this quantization phenomenon, and the quasi-elastic inter-Landau level scattering (QUILLS) model of Heinonen, Taylor, and Girvin [10] and Eaves and Sheard [11], to deduce the maximum electric field experienced by the conducting electrons.

Finally, there has been a puzzle about how inter-Landau level transitions–which in the QUILLS model occur between states distributed across the sample width–can be detected as voltage signals along the sample length. We will give a pictorial explanation of a solution to this puzzle.

## 2. Experiment

### 2.1 Sample and Coordinate System

The sample is a GaAs/Al*_x_*Ga_1−*x*_ As heterostructure grown by molecular beam epitaxy at AT&T Bell Laboratories,^1^ with *x* =0.29 being the fraction of Al atoms replacing Ga atoms in the crystal. The sample is designated as GaAs(8), has a zero magnetic field mobility of about 100 000 cm^2^/(V·s) at 4.2 K, and exhibits excellent integral quantum Hall effect properties. This sample is currently used as a quantized Hall resistance standard to maintain the United States unit of resistance.

**Fig. 1:**
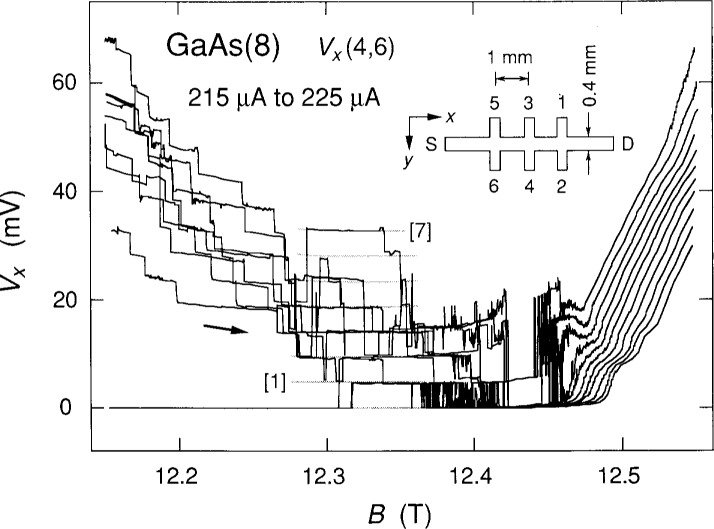
Eleven sweeps of *V_x_*(4,6) versus *B* for the *i* = 2 plateau at 0.33 K with applied currents *I*_SD_ between + 215 μA and + 225 μA in 1 μA increments. The values of *V_x_* generally increase with current. The arrow shows the sweep direction. A family of eight shaded curves is fitted to these data in the vicinity of 12.3 T where the data are current-independent. Voltage quantization numbers are shown in brackets. The inset displays the sample geometry. The actual origin of the coordinate system is located at the source S, halfway across the sample width *w.*

The inset of Fig. 1 shows the sample geometry. It is 4.6 mm long and has a width, *w*, of 0.4 mm. The two outer Hall potential probe pairs are displaced from the central pair by plus and minus 1 mm. The inset also shows the coordinate system. The positive *x* axis points along the sample in the direction of the current *I*_SD_. Note that the conducting charges are *electrons.* The *y* axis points across the sample, and the *z* axis is into the figure for a right-handed coordinate system. The location of the co-ordinte system origin is arbitrary; it is convenient, however, to place it at the source S, and halfway across the sample, so that *−w*/2⩽*y*⩽*w*/2. (This location is not shown in the figure for lack of space.) The magnetic field, *B*, is perpendicular to the sample and points into the figure, in the positive *z* direction. Therefore, in the presence of a *B* field, the electrons enter at the upper left hand corner of the sample and exit at the lower right hand corner. The potential probes 2, 4, and 6 are near the potential of the source S, which is grounded. Probes 1, 3, and 5 are near the drain potential D, and have a positive potential relative to the source.

### 2.2 Longitudinal Voltage Versus Magnetic Field

The dissipative breakdown voltages *V_x_* for this paper were measured between potential probe pair 4 and 6, hereafter denoted as *V_x_* (4,6) *≡ V*(4) − *V*(6). The longitudinal voltage signals on the opposite side of the sample, *V_x_* (3,5), were the same as *V_x_* (4,6), and integer quantum Hall voltages *V*_H_ *= R*_H_*I*_SD_ were observed on probe sets *V*_H_ (3,4) and *V*_H_ (5,6).

Figure 1 shows 11 sweeps of *V_x_* (4,6) versus the magnetic field *B* for the *i* = 2 (12 906.4 Ω) quantized Hall resistance plateau at a temperature of 0.33 K for injected electron currents *I*_SD_ of +215 μA to +225 μA in 1 μA increments, where positive current corresponds to electrons entering the source and exiting the drain. These sweeps are for increasing *B*; similar sweeps were obtained for decreasing *B.* The data clearly show discrete, well-defined, quantized voltage states, with switching between states.

A family of eight equally-spaced shaded curves is also shown in Fig. 1 in the region near 12.3 T where the *V_x_* voltage spacings are nearly current-independent. The curves have equal (quantized) voltage separations at each value of magnetic field, but the voltage separations are allowed to vary slightly with *B* in order to obtain smooth curves that provide the best fit to the data. The eight shaded curves correspond to a *V_x_* = 0.0 mV ground state and seven excited states. Several quantum numbers *M* of the voltage states are labeled in brackets. Note that, over the shaded curve portion of Fig. 1 near 12.3 T, the *M* = 1 transition first occurs at a current of 215 μA, and the *M* = 7 state first appears at 225 μA.

## 3. Analysis

### 3.1 Transition Rates

We first use a simple black-box model [7–9] based on energy conservation arguments to interpret some aspects of the *V_x_* voltage quantization displayed in Fig. 1. A fully-quantized Hall voltage *V*_H_ *= R*_H_*I*_SD_ occurs on probe sets (3,4) and (5,6). A necessary condition of integral *V*_H_ quantization is that allowed eigenstates of the first Landau level are completely filled at those two positions along the sample length and the next Landau level is completely empty. However, there is dissipation in the sample region between those two Hall probe-pair positions, as evidenced by the *V_x_* signal observed on probes (4,6). This dissipation is assumed to arise from transitions in which electrons occupying states of the originally full ground state Landau level are excited to states in higher Landau levels and then return to the lowest Landau level. The electrical energy loss per carrier for *M* Landau level transitions is M*ħω*_c_, where *ω*_c_ = *eB*/m* is the cyclotron angular frequency and m* is the reduced mass of the electron (0.068 times the free electron mass *m*_e_ in GaAs). The power loss is *IV_x_*, and *IV_x_ = r*(2/*i*)*Mħω*_c_, where *r* is the combined transition rate from the ground state to the excited state and then back to the ground state, and *i* is the Hall plateau number. Thus

$$fM=\left(\frac{re}{I}\right)M=\left(\frac{i}{2}\right)\left(\frac{m*}{\hbar }\right)\left(\frac{V_{x}}{B}\right),$$ (1)

where *f* is the ratio of the transition rate *r* within the breakdown region to the rate *I*/*e* that electrons transit the device; *f* can also be interpreted as the fraction of conducting electrons that undergo transitions.

The black-box model predicts that, in the vicinity of *B* = 12.3 T, about 22 % of the conducting electrons are making inter-Landau transitions, with transition rates between 3.0 × 10^14^/s and 3.1 × 10^14^/s for currents between 215 μA and 225 μA.

### 3.2 QUILLS Model

To predict the maximum value of the electric field, *E*_max_, within the sample when breakdown is occurring we use the quasi-elastic inter-Landau level scattering (QUILLS) model of Heinonen, Taylor, and Girvin [10] and Eaves and Sheard [11], and the notation of Cage, Yu, and Reedtz [12]. The conducting electrons have completely filled the maximum allowed number of states of the first (*N* = 0) Landau level, as determined from the charge-carrier surface number density *n*_s_ *= i*(*eB*/*h*). The wavefunctions of the states are represented in the Landau gauge as normalized products of Hermite polynomials across the sample multiplied by plane waves propagating down a length *L_x_* of the sample [12]. In this gauge *A_x_ = −yB_z_* and *A_y_ =A_Z_* = 0, where *A* is the magnetic vector potential. The energy eigenvalue *ϵ_N_* of each state is

$$\epsilon_{N}(y_{0})=(N+\frac{1}{2})\hbar \omega_{c}+ey_{0}E(y_{0})+\frac{1}{2}m*\nu_{x}^{2}(y_{0}),$$ (2)

where

$$y_{0}=(v_{x}/w_{c}+\ell_{B}^{2}k_{x})$$ (3)

is the center of mass position of each state undergoing cycloidal motion, *−w*/2*<y*_0_*<w*/2, *v_x_*(*y*) = *E*(*y*)/*B* is the electron drift velocity down the sample, *ℓ_B_* = (*ħ*/*eB*)^1/2^ is the magnetic length, and *k_x_* = 2*πN_k_*/*L_x_* is the wavevector for the state located at position *y*_0_ with an associated positive or negative integer quantum number *N_k_.* The eigenstates are represented by the quantum numbers (*N,N_k_*), and the wavefunction for each state is

$$\begin{array}{c} \psi_{N,N_{k}}(x,y)=\frac{1}{{\left(L_{x}\right)}^{1/2}}e^{i2\pi N_{k}x/L_{x}}\frac{1}{{\left(2^{N}N!\right)}^{1/2}}\frac{1}{{\left(\pi \ell_{B}^{2}\right)}^{1/4}}\\ e^{-{\left(y-y_{0}\right)}^{2}/2\ell_{B}^{2}}H_{N}[(y-y_{0})/\ell_{B}], \end{array}$$ (4)

where *H_N_*[(*y* − *y*_0_)/*ℓ_B_*] is a Hermite polynomial.

**Fig. 2:**
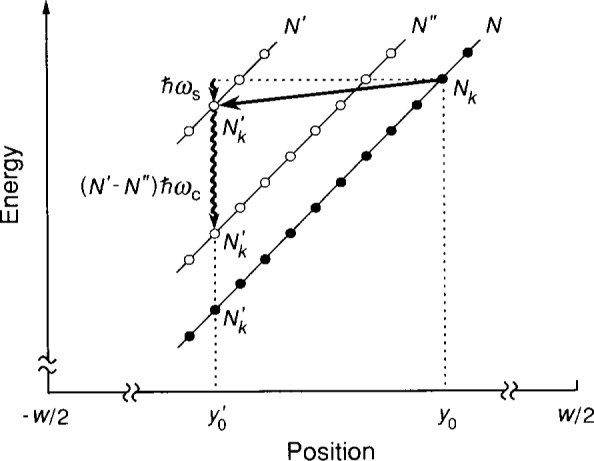
Total energy eigenvalues *ϵ_N_*(*y*), represented as circles, as a function of position *y* across a region of the sample width *w* for Landau levels *N, N*′, and an intervening level *N".* The eigenvalues have unique quantum numbers (*N,N_k_*). Initially occupied eigenstates are indicated by solid circles, empty states by open circles. The figure shows a QUILLS transition from the eigenstate at position *y*_0_ in level *N* to position $y_{0}^{\prime }$ in level *N*′, and an associated acoustic phonon of energy *ħω_s_.* The decay back to the ground state, either directly or through an intermediate level *N"*, is also shown, along with its associated optical phonon.

Figure 2 displays the energy eigenvalues as circles for the allowed states of Landau levels *N* and *N′*, plus an intervening level *N"*, over a portion of the sample width in which the average value of the electric field is *E*(*y*) = −∇*V*(*y*) = −Δ*V*(*y*)/Δ*y*. The change in energy over the region Δ*y* is Δ*ϵ*(*y*) = *q*Δ*V*(*y*) *= −e*Δ*V*(*y*). Therefore

E(y)=Δϵ(y)/eΔy (5)

is the slope of a Landau level at position *y* divided by the charge *e.* The initially occupied eigenstates are indicated in the figure by solid circles, empty states by open circles. The spatial separation between adjacent states is $\Delta y_{0}=2\pi \ell_{B}^{2}/L_{x}$ and the energy separation between adjacent Landau levels is *ħω_c_.*

If the electric field *E*(*y*) becomes sufficiently large then the Landau levels tilt enough to allow a population inversion, and electrons occupying eigenstates (*N,N_k_*) at positions *y*_0_ in the lowest Landau level *N* can make transitions to states $\left(N^{\prime },N_{k}^{\prime }\right)$ of lower total energy at positions $y_{0}^{\prime }$ in a higher Landau level *N*′. In order to conserve energy and momentum, acoustic phonons of energy *ħω_s_ = ħv_s_K_x_* are emitted in the *x* direction during these transitions, where *v*_s_ is the velocity of sound (2.47 × 10^3^ m/s in GaAs [13] and $K_{x}=2\pi (N_{k}-N_{k}^{\prime })/L_{x}=(N^{\prime }-N)\omega_{c}/(v_{x}-v_{s})$ is the acoustic phonon wavevector. After emitting the acoustic phonons, the electrons then emit optical phonons of total energy (*N*′ *− N*)*ħω_c_* and return to eigenstates of the initial Landau level *N.* These optical phonon transitions could occur either directly from the *N*′ Landau level to unoccupied states in the lowest Landau level *N*, or via a sequence of one or more intermediate Landau levels *N".*

It would appear in Fig. 2 that there are no unoccupied ground states for the excited electrons to decay into, but in reality unoccupied states exist because, as will be explained in Sec. 5, the ground state electrons continuously redistribute themselves in order to maintain the electric field whenever electrons undergo acoustic phonon transitions into the *N*′ Landau level.

### 3.3 Maximum Electric Field

The physics of these QUILLS transitions from eigenstates in the lowest Landau level to states in a higher Landau level, and then back down to states in the lowest level is obviously very complicated and nonlinear. One would have to calculate the matrix elements of both the acoustic and the optical phonon transitions to properly model the process. We overcame this problem in Sec. 3.1 by using a black-box model to obtain the transition rates. We can also obtain a reasonable estimate of the maximum electric field by noting that: (a) the spatial extent of the *y*-axis motion of the wavefunction described in Eq. (4) decays rapidly beyond the turning points of a classical harmonic oscillator whose amplitude of motion is $A_{N}=\ell_{B}\sqrt{2N+1}$ [14], where *ℓ_B_* = (*ħ*/*eB*)^1/2^ is 7.3 nm at 12.3 T; and (b) the matrix elements of the acoustic phonon transitions become significant only when the initial and final state wavefunctions overlap [11,12]. Transitions between the *N* and *N*′ eigenstates therefore commence when

$$(y_{0}-y_{0}^{'})\approx \ell_{B}\left(\sqrt{2N+1}+\sqrt{2N^{\prime }+1}\right),$$ (6)

where in our case *N* = 0 for the *i* = 2 plateau, *M = N*′ *− N = N*′, and the scale factor is of order 1. The maximum electric field can now be obtained from Eqs. (5) and (6) and Fig. 2. It is

$$E_{\max}\approx \frac{M\hbar \omega_{c}}{e(y_{0}-y_{0}^{\prime })},$$ (7)

where we have neglected the small contribution *ħω*_s_ due to the acoustic phonon transition in the numerator of Eq. (7).

**Fig. 3:**
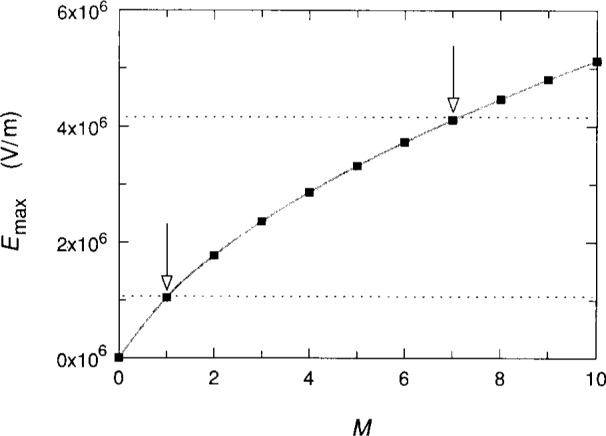
Values of the electric field *E*_max_ versus quantum numbers *M* obtained using Eqs. (6) and (7) at *B* = 12.3 T. The shaded curve is to guide the eye. The two dotted lines are the values of *E*_max_ chosen for the maximum electric fields observed in the *M* = 1 and *M* = 7 transitions of Fig. 1. They are 1.1 × 10^6^ V/m and 4.2 × 10^6^ V/m, respectively.

Figure 3 shows the values of *E*_max_ versus *M* obtained using Eqs. (6) and (7) at *B* = 12.3 T. The two dotted lines indicate the values of the maximum electric fields chosen for the *M* = 1 and *M* = 7 transitions of Fig. 1 at 215 μA and 225 μA currents, respectively. The values are 1.1 × 10^6^ V/m and 4.2 × 10^6^ V/m; they are made slightly larger than the calculated values of 1.05 × 10^6^ V/m and 4.11 × 10^6^ V/m to assure acoustic phonon transitions. It was possible to obtain these values of *E*_max_ only because the *M* values could be uniquely identified in the breakdown data of Fig. 1.

## 4. Discussion

We have used a QUILLS model to determine the maximum electric fields at two applied currents. Now that the electric fields are known we can calculate the values of other parameters–and then speculate about the location of the breakdown region and where the energy is dissipated.

### 4.1 Calculation of other Parameters

The maximum electric field values of 1.1 × 10^6^ V/m and 4.2 × 10^6^ V/m obtained in the previous section generate large local current densities: *J_x_* = *σ_xy_E*_max_ *= E*_max_/12 906.4 Ω = 85 A/m and 325 A/m, respectively at *I*_SD_ = 215 μA and 225 μA. The electron drift velocities in this region of the sample are *v_x_ = E*_max_/*B* = 8.9 × 10^4^ m/s and 3.4 × 10^5^ m/s. These electron velocities are 36 and 138 times faster than the acoustic phonon velocities. The values of the acoustic phonon energies *ħω_s_* = *ħMω_c_v_s_*/(*v_x_ – v*_s_) are 2.9 % and 0.7 *%* of the total optical phonon energies *Mħω_c_* (which are 3.4 × 10^−21^ J and 2.4 × 10^−20^ J, respectively).

### 4.2 Location of Breakdown Region

Where is *E*_max_ located within the sample? Fontein et al. [15] have made contactless measurements of the potential distributions throughout quantum Hall samples using the electro-optic Pockels effect. They observed major contributions to the Hall voltage near the sample sides. That is reasonable because a confining potential with large gradients exists along the sample periphery. There is also a logarithmic charge-redistribution potential due to an equilibrium between the Lorentz and Coulomb forces on the conducting electrons [16–20] that also increases dramatically at the sample sides. The breakdown region where *E*_max_ occurs is therefore likely to be near the side where electrons are deflected; in our case this is near potential probes 4 and 6.

### 4.3 Energy Dissipation

Energy is dissipated within the sample during breakdown, as evidenced by the large *V_x_* signals in Fig. 1. However, this energy cannot be significantly dissipated within the breakdown region itself because the *V_x_* signals are quantized. Local heating in the breakdown region would thermally excite electrons out of the ground state eigenenergies shown in Fig. 2 into higher Landau levels, and the quantized *V_x_* signal would be washed-out. If QUILLS is the appropriate mechanism to describe the breakdown, then the acoustic and optical phonons must transmit the energy away from the breakdown region and then dissipate it elsewhere – most likely at the sample periphery or into the GaAs crystal.

## 5. Detection of Longitudinal Voltage Signals

**Fig. 4:**
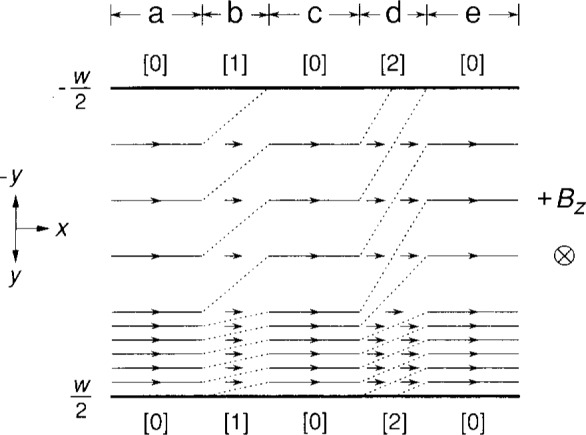
Schematic drawing showing why QUILLS transitions across the sample are observed as quantized voltages along the sample. Breakdown is occurring in the high electric field regions of sections b and d at this instant of time. The conducting electrons completely occupy the ground state eigenenergies in sections a, c, and e. See Sec. 5 for details.

There remains the question of why QUILLS transitions *across* the sample can be detected as quantized voltages *along* the sample. Figure 4 provides a pictorial explanation that involves electric fields in the transverse *y* direction and voltage increases in the longitudinal *x* direction. For simplicity, the schematic drawing shows only two values of electric field across this portion of the sample. In reality there is a wide range of *E* values.

The electric field strengths are indicated by the spacings between equipotential lines. The choice of equipotential spacing is arbitrary. In Fig. 4 we choose the longitudinal voltage quantization *V_x_*/*M* = (2/*i*)*fħω*_c_/*e* as the spacing. The value of *V_x_*/*M* depends on the fraction *f*, and would be equal to *ħω*_c_/*e* for the *i* = 2 plateau if *f* was 100 %. It is about 4.6 mV for the data in the shaded curve portion of Fig. 1 where *f* is about 22 %. The Hall voltage is *V*_H_ = (*n +* 1)*V_x_* for this choice of spacing, where *n* is the number of equipotential lines within the sample interior. There would be 616 equipotential lines across the sample for the present data at 220 μA. For clarity, Fig. 4 shows only 9 of these lines.

The highest electric field value, *E*_max_, could be anywhere across the sample. In the following discussion we assume it is near the side of the sample where conducting electrons are deflected by the Lorentz force. This high electric field covers a significant fraction of the sample width in the figure, but would occupy a narrow region in an actual sample. If *E*_max_ is large enough then QUILLS can occur, such as the *M* = 1 and *M* = 2 transitions indicated at this instant of time in sections b and d of Fig. 4.

Electrons in the QUILLS model physically move from eigenstate positions *y*_0_ to positions $y_{0}^{\prime }$ during breakdown – all the while redistributing themselves in the *y* direction to maintain a constant electric field. We see in Fig. 2 that, for electrons making QUILLS transitions with our current and magnetic field directions, the electrons move to more negative values of *y;* their ground eigenstate energies *decrease;* and their potentials *increase* because Δ*V*(*y*) = *−*Δ*ϵ*(*y*)/*e.*

QUILLS transitions occur only in high electric field regions. The QUILLS process is complicated, and we do not know the actual shape of the equipotentials when QUILLS transitions are occurring in regions such as b and d of Fig. 4. However, the potential increases each time there is a transition – and the transitions probably occur along a finite length of the sample. So the equipotentials in the high electric field breakdown regions are indicated simply as dotted straight lines. This breakdown phenomenon causes a charge-redistribution to smaller values of *y* – which results in additional Coulomb repulsion of electrons in the low electric field regions, thereby altering their potential distributions. We indicate this by also using dotted straight lines in the low field areas of regions b and d.

Some dotted lines intersect the sample sides, causing the potentials to increase along the sample periphery. The change in potential is equal on both sides of the sample, in agreement with experiment. The electrons have all returned to the ground eigenstates in sections c and e. Therefore the equipotentials must be parallel to the sample length, as they were originally in section a. The *y*-components of *E* in the high electric field regions are constant along the sample in Fig. 4, whether or not breakdown is occurring. This condition is required in the QUILLS model.

Arrows indicate the direction of electron flow–which is directly down the sample since we have not included any localized regions of significant extent, and the shifts in *y* positions $\Delta y=y_{0}^{\prime }-y_{0}\approx -M\hbar \omega_{c}/eE_{\max}$ during QUILLS transitions are too small to be shown in Fig. 4. For example, Δ*y* is −0.019 μm for the *M* = 1 transition at 1.1 × 10^6^ V/m and −0.035 μm for the *M* = 7 transition at 4.2 × 10^6^ V/m, respectively. The current density *J_x_ = σ_xy_E_y_* is largest in the high electric field regions.

Conducting electrons move along equipotential lines in ground state sections a, c, and e. No *V_x_* signals arise from these regions on potential probes placed along either side of the sample. The conducting electrons cross equipotential lines in breakdown regions b and d, and *V_x_* signals occur. We note that if the potential probes were placed between sections e and a of Fig. 4 then the *V_x_* signal at this instant in time would be three times larger than if the probes were between sections c and a. This observation illustrates that the *M* values deduced from the *V_x_* signal could be due to one region of [0] to [*M*] QUILLS transitions, *M* regions of [0] to [1] transitions, or an intermediate combination – such as the [0] to [1] and [0] to [2] regions shown in Fig. 4. If, however, the *V_x_* signal is due to multiple breakdown regions then the fraction *f* of electrons making the transitions in each region must be nearly equal – otherwise the voltage quantization *V_x_/M* would be washed-out. It is therefore likely that the data of Fig. 1 is due to a single breakdown region since we often observe different values of *f* or different critical current onset values for breakdown when using other *V_x_* probe sets along this sample.

## 6. Conclusions

The maximum value of the electric field across the width of a quantum Hall effect sample can be estimated if the sample is in the breakdown regime and the quantized longitudinal voltage signal has a rich enough structure to enable the quantum numbers to be uniquely identified. A QUILLS model is used to calculate the electric field. A pictorial description can explain the puzzle of how QUILLS transitions occurring between eigenstates distributed across the sample width can be detected as voltage signals along the sample length.

## Footnotes

1 Certain commercial equipment, instruments, or materials are identified in this paper to foster understanding. Such identification does not imply recommendation or endorsement by the National Institute of Standards and Technology, nor does it imply that the materials or equipment identified are necessarily the best available for the purpose.
